# Retrograde Endoscopic Management of Completely Transected Ureter Discovered Postoperatively

**DOI:** 10.1089/cren.2018.0029

**Published:** 2018-06-01

**Authors:** Dong Wang, Shaw P. Wan

**Affiliations:** ^1^ The People's Hospital of Huantai, Zibo City, Shandong, China.; ^2^ Department of Urology, The First People's Hospital of Xiaoshan District, Hangzhou, Zhejiang, China.

**Keywords:** ureteral injury, stenting, delayed diagnosis

## Abstract

Inadvertent injury of the ureter is a known risk of pelvic surgery. If the injury is noticed intraoperatively, the treatment is relatively straightforward. However, if the discovery of the injury is delayed, the treatment is more difficult and less assured. We encountered a case of a completely transected ureter that had occurred during laparoscopic sigmoid colectomy and was diagnosed on the 8th postoperative day. The patient was treated with minimally invasive retrograde endoscopic realignment with excellent results. Therefore, we decided to report this case and perform a literature review on this subject.

## Introduction

Inadvertent injury of the ureter is a known risk of pelvic surgery. The incidence of ureteral injury in colorectal resection was reported to be between 0.11% and 0.24%.^1,2^ Laparoscopic procedures have a higher incidence of ureteral injury than open procedures.^2^ If the injury is discovered intraoperatively, the treatment is relatively straightforward. However, if the injury is discovered in a delayed scenario, the treatment is more difficult and contentious. We encountered a completely transected ureter that had occurred during laparoscopic sigmoid colectomy and was discovered on the 8th postoperative day. We treated it with retrograde endoscopic stenting only and achieved excellent results; hence this report and a review of the literature on this subject. This case report is written with the explicit consent from the patient and approval from our Institutional Ethics Committee.

## Case Report

**FIG. 1.**
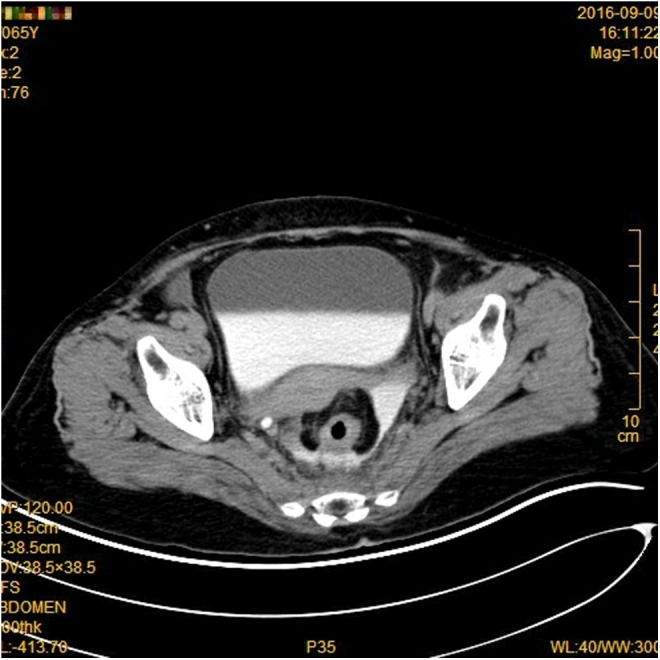
Contrast extravasation in the pelvis and around the descending colon.

**FIG. 2.**
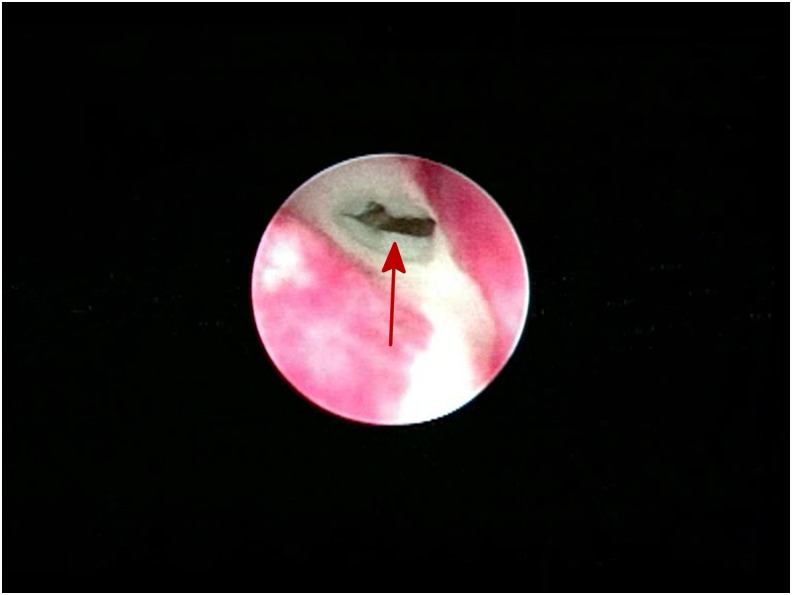
The distal end of the transected ureter.

**FIG. 3.**
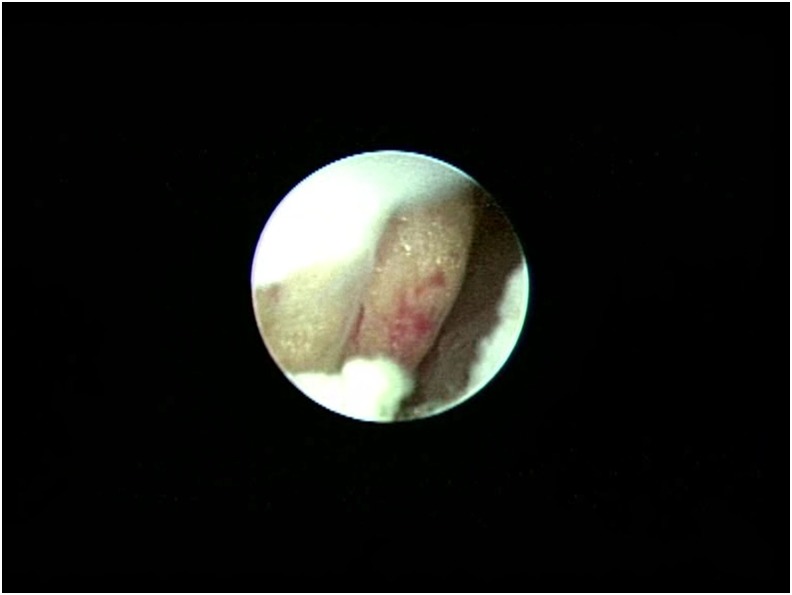
Intraabdominal cavity seen through the distal end of the transected ureter.

**FIG. 4.**
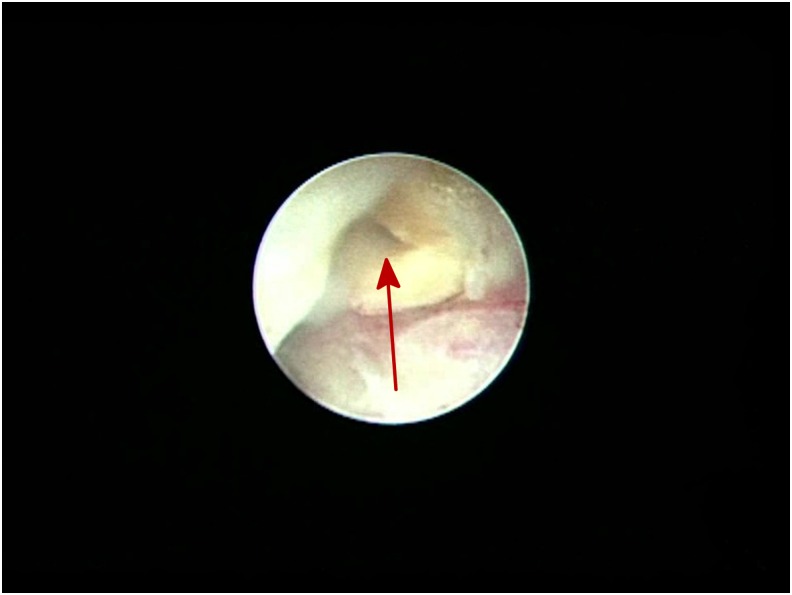
The proximal end of the transected ureter.

The patient was a 65-year-old woman who presented with a 1-month history of bloody stool. A digital colonoscopy with biopsies revealed adenocarcinoma in the sigmoid colon. The patient elected to undergo primary laparoscopic colon resection, and the procedure was reported to be uneventful. However, on the 6th postoperative day, the patient noticed a large amount of yellow fluid coming out of a left side abdominal drain. The fluid appeared to be urine. An abdominal ultrasonography showed a collection of fluid in the patient's pelvis. A contrasted computed tomography (CT) scan showed contrast extravasation in the pelvis and around the descending colon (Fig. 1). The patient had decreased serum protein and albumin; however, complete blood count, creatinine, liver functions, and urine analysis were normal. On the 8th postoperative day, the patient was taken to the operating room for a ureteroscopy. The ureteroscopy revealed that the left ureter was completely severed about 4 to 5 cm from the ureteral orifice (Fig. 2, transected distal end of the ureter). The bowels could be seen through the ureteroscope (Fig. 3, intraabdominal cavity with bowels seen through the distal end of the transected ureter). No other obvious injury was identified. With patience, persistence, and some difficulty, the severed upper end of the ureter was identified and entered (Fig. 4, the proximal end of the transected ureter). We estimated that there was a 3- to 4-cm gap between the two ends of the ureter. Two 0.035″ guidewires were first passed, followed by the placement of two 4.5F Double-J ureteral stents. After placing the Double-J stents, the abdominal drainage quickly subsided. An abdominal ultrasonography 6 days after tube placement showed complete resolution of the abdominal fluid collection. The abdominal drain was removed and the patient was discharged. At the 3-month follow-up, a repeat CT scan showed no hydronephrosis, no abdominal fluid collection, and no contrast extravasation. The patient, however, had an asymptomatic urinary tract infection from *Klebsiella pneumoniae*, which was treated and resolved. A follow-up ureteroscopy over a guidewire showed excellent healing and realignment of the disrupted ureter. The only obvious sign of the transacted ureter was that the mucosa was paler than normal. Because of this unconventional treatment, we decided to continue stenting the ureter with two fresh 4.5F Double-J stents for an additional 4 weeks. Stents were removed 4 months after the injury. Follow-up contrast-enhanced CT scans taken 8 and 14 months after the initial endoscopic treatment showed mild but unchanged residual dilation of the renal pelvis and ureter with good drainage. There were no other abnormalities.

## Discussion

Intraoperative iatrogenic injuries of the ureter can occasionally occur during pelvic surgery. Some surgeons routinely request the placement of ureteral catheters during excisions of large pelvic masses or radical resections of a malignant organ to aid in identifying ureters. When a major ureteral injury is recognized during or shortly after the procedure, an immediate repair of the major injury, either by an end-to-end anastomosis or by ureteral neocystostomy, is recommended. For a lesser injury, ureteral stenting will often suffice. However, when an injury is discovered several days postoperatively, the treatment is more difficult. The inflammation of the injured ureter and the surrounding tissue will severely hinder recovery. In our patient, the ureter was probably inadvertently seared with the harmonic scalpel. Several days later, the necrosis of the injured ureter resulted in complete disruption of the ureter and free drainage of urine into abdominal cavity. At this point, we felt that the patient was not in the optimal condition for primary repair. After counseling the patient, we decided to try an endoscopic approach. If the more conservative approach failed, we planned to immediately switch to a primary repair method, probably an end-to-end anastomosis with mobilization of the kidney or a reimplantation using a Boari's flap.

It is worthwhile to mention that searching for the proximal end of the disrupted ureter was a painstaking task. It required patience, persistence, and possibly some element of luck. We decided to place two smaller Double-J ureteral stents rather than a single larger size stent, as it has been reported that two smaller stents seem to offer better drainage and result in a lower incidence of ureteral stricture than a single larger stent.^3,4^ We left the ureteral stents in place for 4 months and changed them 3 months poststenting. This was because of our uncertainty of this unorthodox management. In retrospect, a shorter period of the indwelling stents would have sufficed. Fortunately, our patient had an excellent outcome, hence this report. This procedure might be a reasonable first venue to be considered for other patients with similar clinical situations in the future.

Endoscopic management of a completely transected ureter discovered postoperatively is not entirely novel. Liu et al.^5^ described the management of completely transected ureters discovered postoperatively in eight patients. They employed a method combining antegrade flexible ureteroscopy and retrograde rigid ureteroscopy to re-establish the continuity and placement of ureteral stents. Three 5F Double-J stents were placed in each patient. They reported 100% success in re-establishing ureteral continuity. However, two out of eight patients developed ureteral stricture.

## Conclusion

We report a case of effective retrograde endoscopic management of a completely transected ureter that was discovered 8 days postoperatively. This case illustrates that this approach is feasible and can be effective, and, therefore, should be considered before more invasive treatments. Nevertheless, it should be emphasized that primary repair of completely transected ureters remains the standard of care for such injuries.

## Abbreviation Used

CT: computed tomography

## References

[1] Eswara JR, Raup VT, Potretzke AM, et al. Outcomes of iatrogenic genitourinary injuries during colorectal surgery. Urology 2015;86:1228–1233 26368509 10.1016/j.urology.2015.06.065

[2] Palaniappa NC, Telem DA, Ranasinghe NE, et al. Incidence of iatrogenic ureteral injury after laparoscopic colectomy. Arch Surg 2012;147:267–271 22430909 10.1001/archsurg.2011.2029

[3] Liu JS, Hrebinko RL The use of 2 ipsilateral ureteral stents for relief of ureteral obstruction from extrinsic compression. J Urol 1998;159:179–181 9400466 10.1016/s0022-5347(01)64050-3

[4] Rotariu P, Yohannes P, Alexianu M, et al. Management of malignant extrinsic compression of the ureter by simultaneous placement of two ipsilateral ureteral stents. J Endourol 2001;15:979–983 10.1089/089277901317203047 11789979

[5] Liu C, Zhang X, Xue D, et al. Endoscopic realignment in the management of complete transected ureter. Int Urol Nephrol 2014;46:335–340 23925502 10.1007/s11255-013-0535-7

